# Association of Abnormal Glucose Metabolism and Inflammation With Prognosis in Patients With Acute Coronary Syndrome

**DOI:** 10.1111/1753-0407.70134

**Published:** 2025-07-30

**Authors:** Chong Zhang, Wenjin Peng, Tianqi Wang, Meng Ning, Yi Chen, Yingwu Liu

**Affiliations:** ^1^ Neuroscience Intensive Care Unit The Second Affiliated Hospital of Zhejiang University School of Medicine Hangzhou Zhejiang China; ^2^ The Third Central Clinical College of Tianjin Medical University Tianjin China; ^3^ Tianjin Key Laboratory of Extracorporeal Life Support for Critical Diseases Tianjin China; ^4^ Artificial Cell Engineering Technology Research Center Tianjin China; ^5^ Tianjin Institute of Hepatobiliary Disease Tianjin China; ^6^ Department of Heart Center The Third Central Hospital of Tianjin Tianjin China; ^7^ School of Medicine Nankai University Tianjin China

**Keywords:** acute coronary syndrome, inflammatory markers, major adverse cardiovascular events, stress hyperglycemia ratio, triglyceride‐glucose

## Abstract

**Background:**

In acute coronary syndrome (ACS) patients, the relationship between abnormal glucose metabolism markers, such as the triglyceride‐glucose (TyG) index and stress hyperglycemia ratio (SHR), and inflammatory markers remains unclear. Furthermore, their interaction and impact on long‐term prognosis have yet to be investigated in clinical cohorts.

**Methods:**

In this study, K‐means clustering was performed on the TyG index, SHR, and inflammatory markers, including high mobility group box‐1 protein, platelet‐derived growth factor, phenylacetylglutamine, lysophosphatidic acid, and citrullinated histone H3. A Cox proportional hazards model was used to assess the association between cluster phenotypes and 1‐year major adverse cardiovascular events (MACE) risk in ACS patients.

**Results:**

Among 363 ACS patients, 62 developed MACE during the 1‐year follow‐up. SHR correlated positively with the TyG index and inflammatory markers. K‐means clustering identified two phenotypes: normal glucose metabolism/low inflammation and abnormal glucose metabolism/high inflammation. Multivariable Cox analysis showed the latter was strongly linked to MACE (adjusted hazard ratio: 3.84, 95% confidence interval: 1.93–7.64), and early guideline‐directed medical therapy reduced MACE risk in this high‐risk group.

**Conclusions:**

ACS patients with abnormal glucose metabolism and high inflammation have a higher long‐term MACE risk than those with normal glucose metabolism and low inflammation. Early guideline‐directed medical therapy, alongside anti‐inflammatory therapy and hypoglycemic agents, may improve long‐term outcomes in this high‐risk group.

## Graphical abstract

Inflammation, abnormal glucose metabolism, and MACE. Abbreviations: ACS, acute coronary syndrome; CH3, citrullinated histone H3; CI, confidence interval; GDMT, guideline‐directed medical therapy; GRACE, Global Registry of Acute Coronary Events; HMGB1, high mobility group box‐1 protein; HR, hazard ratio; LPA, lysophosphatidic acid; MACE, major adverse cardiovascular events; PAGln, phenylacetylglutamine; PDGF, platelet‐derived growth factor; SHR, stress hyperglycemia ratio; TyG, triglyceride‐glucose.

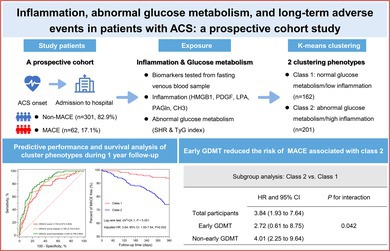

**Abbreviations**
ACS: acute coronary syndrome
CH3: citrullinated histone H3
CI: confidence interval
GDMT: guideline‐directed medical therapy
GRACE: global registry of acute coronary events
HMGB1: high mobility group box‐1 protein
HR: hazard ratio
LPA: lysophosphatidic acid
MACE: major adverse cardiovascular events
PAGln: phenylacetylglutamine
PDGF: platelet‐derived growth factor
SHR: stress hyperglycemia ratio
TyG: triglyceride‐glucose

**Summary**

- The complex interplay between abnormal glucose metabolism markers and inflammatory markers in acute coronary syndrome (ACS) patients, and how these interactions impact long‐term cardiovascular outcomes remains unknown.
- ACS patients with abnormal glucose metabolism/high inflammation experienced a significantly increased risk of major adverse cardiovascular events (MACE) during 1‐year follow‐up.
- Early guideline‐directed medical therapy could mitigate the MACE risk associated with abnormal glucose metabolism/high inflammation in patients with ACS.

## 1 Introduction

Recent studies indicate that glucose metabolism abnormalities not only disrupt energy balance but also play a key role in inflammation regulation [1, 2]. In acute coronary syndrome (ACS), abnormal glucose metabolism interacts with inflammatory mediators, driving endothelial dysfunction, accelerating atherosclerosis progression, and increasing the risk of adverse events. Previous studies found that stress hyperglycemia ratio (SHR) and triglyceride‐glucose (TyG) index, indicators of abnormal glucose metabolism and stress hyperglycemia, were associated with a higher major adverse cardiovascular events (MACE) risk in ACS patients [3, 4, 5, 6]. Under hyperglycemia, inflammatory mediators such as high mobility group box‐1 protein (HMGB1), platelet‐derived growth factor (PDGF), phenylacetylglutamine (PAGln), lysophosphatidic acid (LPA), and citrullinated histone H3 (CH3) are upregulated, yet research on their specific roles remains limited. These mediators not only modulate local inflammation but may also propagate systemic effects through long‐range signaling pathways.

A key mechanism in this process is oxidative stress, which increases sharply in hyperglycemia, activating inflammatory pathways such as nuclear factor‐κB. HMGB1, a critical danger‐associated molecular pattern, is released in response to oxidative stress and binds to the receptor for advanced glycation end products (RAGE), creating a positive feedback loop that amplifies local and systemic inflammation [7]. Meanwhile, PDGF is significantly upregulated in hyperglycemia, promoting vascular smooth muscle cell proliferation and migration, accelerating intimal hyperplasia, and destabilizing atherosclerotic plaques, laying the molecular foundation for ACS onset and its complications.

Moreover, PAGln, a gut microbiota‐derived metabolite, fluctuates in hyperglycemia, linking glucose metabolism abnormalities to systemic inflammation through gut microbiota modulation [8, 9]. LPA, a bioactive lipid, promotes vascular cell proliferation, migration, platelet activation, and inflammatory mediator release, shaping the local inflammatory environment [10, 11]. CH3, a neutrophil extracellular traps formation marker, may connect inflammation to glucose metabolism dysregulation [12], offering new insights into ACS pathogenesis.

This study uses a prospective cohort design with quantitative analysis of inflammatory mediators and glucose metabolism biomarkers aim to explore their impacts on long‐term MACE in ACS patients.

## 2 Methods

### 2.1 Study Patients

**FIGURE 1:**
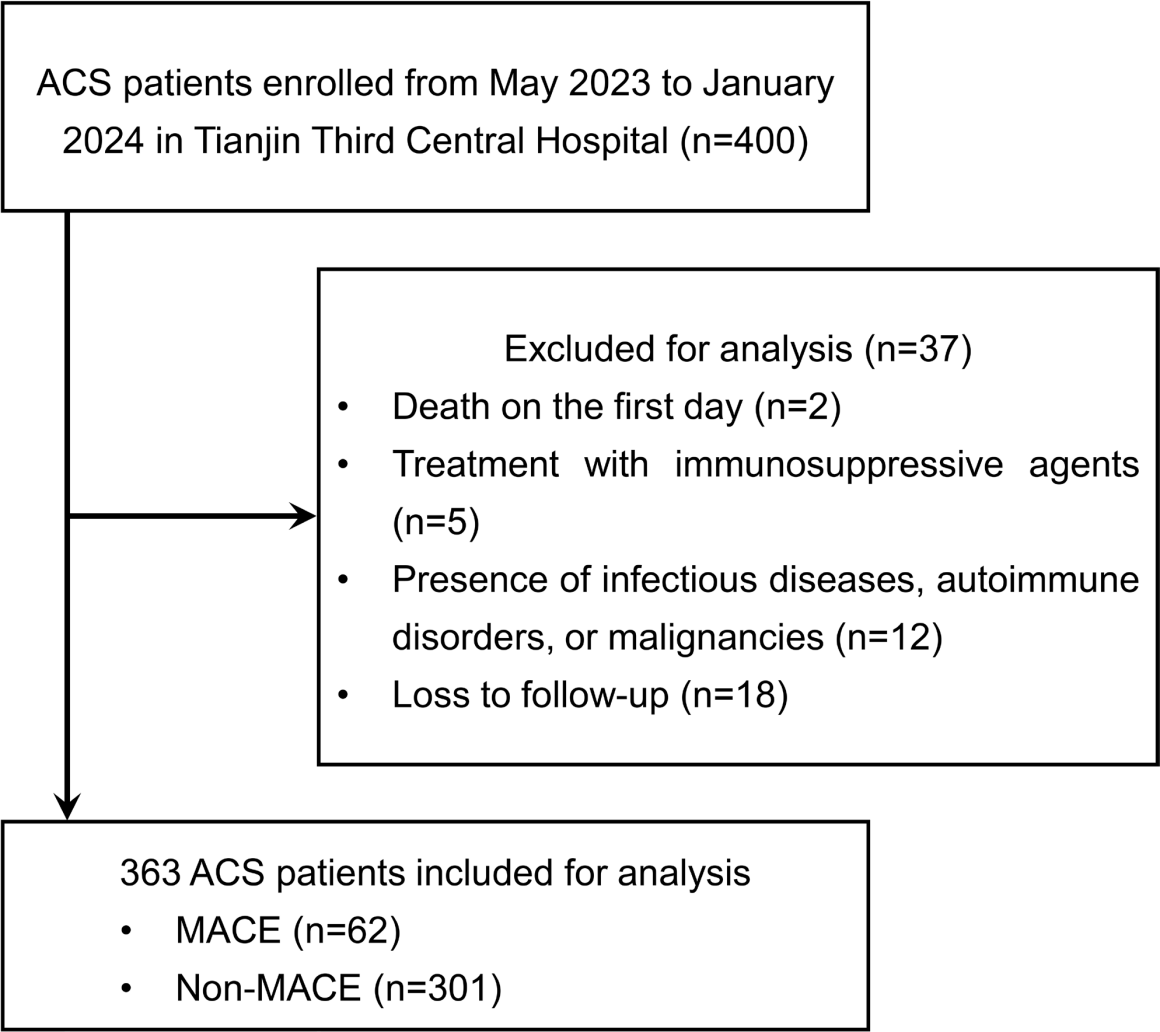
Study flow chart. ACS, acute coronary syndrome; MACE, major adverse cardiovascular events.

This study is a single‐center prospective cohort study approved by the Ethics Committee of Tianjin Third Central Hospital (IRB2023‐015‐01). Written informed consent was obtained from all patients before enrollment. Between May 2023 and January 2024, a total of 400 patients aged 18 years or older with ACS were enrolled at the Heart Center of Tianjin Third Central Hospital. The exclusion criteria were as follows: death on the first day of admission, treatment with immunosuppressive agents, presence of infectious diseases, autoimmune disorders, or malignancies, and loss to follow‐up. Ultimately, 363 ACS patients were included in the final analysis (Figure 1).

### 2.2 Extraction of Inflammatory Biomarkers

Plasma levels of HMGB1, PDGF, PAGln, LPA, and CH3 were quantified using enzyme‐linked immunosorbent assay kits according to the manufacturer's instructions. Fasting venous blood samples (5 mL) were collected within 24 h of admission using ethylene diamine tetraacetic acid anticoagulant tubes, then centrifuged at 2000 rpm for 10 min at 4°C to separate plasma. The supernatant was aliquoted and stored at −80°C until batch analysis.

### 2.3 Definition of Outcomes and Covariables

The primary endpoint was the first occurrence of MACE within 1 year of follow‐up. Follow‐up was conducted monthly via phone calls, outpatient visits, or hospitalization records. MACE was defined as a composite endpoint comprising all‐cause mortality, recurrent myocardial infarction, heart failure, ischemic stroke, and malignant arrhythmias (including ventricular tachycardia, ventricular fibrillation, and cardiac arrest).

The covariables in this study include: (1) demographics (age, sex, smoking history, admission diagnosis); (2) medical history (diabetes mellitus, hypertension, hyperlipidemia, myocardial infarction, prior percutaneous coronary intervention [PCI], atrial fibrillation, heart failure, chronic obstructive pulmonary disease); (3) biochemical markers (white blood cell count, neutrophil count, lymphocyte count, monocyte count, platelet count, hemoglobin, systemic inflammation response index [SIRI], fasting blood glucose [FBG], hemoglobin A1c [HbA1c], triglycerides [TG], low‐density lipoprotein cholesterol, high‐density lipoprotein cholesterol, SHR, TyG, troponin I, HMGB1, PDGF, LPA, PAGln, CH3); (4) vital signs (systolic blood pressure, diastolic blood pressure, heart rate, Killip classification); (5) left ventricular ejection fraction (LVEF); (6) treatment strategies within the first 24 h (aspirin, P2Y_12_ receptor inhibitors, β‐blockers, statins, angiotensin‐converting enzyme inhibitor/angiotensin receptor blocker [ACEI/ARB], guideline‐directed medical therapy [GDMT], anticoagulants, and PCI) were recorded. TyG was calculated as: Ln [FBG (mg/dL) × TG (mg/dL)]/2 [4]. SHR was calculated as: FBG (mg/dL)/[28.7 × HbA1c (%) − 46.7] [13]. SIRI, a composite index incorporating key leukocyte subtypes, has been validated as a marker of systemic inflammation in ACS patients and is calculated as: [neutrophil count (10^9^) × monocyte count (10^9^)]/lymphocyte count (10^9^) [14, 15]. GDMT was defined as the combination of aspirin/P2Y_12_ receptor inhibitors, β‐blockers, statins, and ACEI/ARB within the first 24 h on admission [16].

### 2.4 Statistical Analysis

Continuous variables were expressed as mean ± SD or median (interquartile range) as appropriate, while categorical variables were presented as counts and percentages. Baseline characteristics were compared using the Wilcoxon rank‐sum test, *t*‐test, or chi‐square test.

Based on biological mechanisms and the observation of significantly elevated levels of SHR, TyG index, HMGB1, PDGF, LPA, PAGln, and CH3 in the MACE group versus the non‐MACE group, we applied K‐means clustering (using Euclidean distance) to classify these biomarkers, optimizing cluster numbers via the elbow method and silhouette coefficient. Covariables with significant baseline differences between MACE and non‐MACE groups were included in adjusted models. Kaplan–Meier and Cox models assessed the association between cluster phenotypes and 1‐year MACE risk. Receiver operating characteristic (ROC) curves—area under curve (AUC) analysis evaluated whether clustering improved the Global Registry of Acute Coronary Events (GRACE) score's predictive performance. Subgroup analysis explored associations stratified by age, sex, LVEF, history of acute myocardial infarction, history of PCI, early GDMT therapy, and PCI treatment. Analysis was conducted using Stata 15.1 (StataCorp, USA) and R 4.4.2 (R Foundation, Austria), with *p* < 0.05 considered statistically significant.

## 3 Results

### 3.1 Baseline Characteristic

A total of 363 ACS patients were included, of whom 62 (17.1%) experienced MACE during 1 year follow‐up. Compared to those without MACE, patients in the MACE group had a higher Killip classification, a greater proportion of ST‐segment elevation myocardial infarction (STEMI) patients, and elevated levels of SHR, TyG index, HMGB1, PDGF, LPA, PAGln, and CH3. Additionally, they had lower LVEF (Table 1).

**TABLE 1:** Baseline characteristics between MACE and non‐MACE groups.

Variables	Non‐MACE, *n* = 301	MACE, *n* = 62	*p*
Demographic			
Age, years	67.5 ± 13.6	65.5 ± 16.9	0.32
Male, *n* (%)	177 (58.8)	39 (62.9)	0.55
STEMI, *n* (%)	104 (34.6)	36 (58.1)	< 0.001
Smoking, *n* (%)	143 (47.5)	38 (61.3)	0.048
Medical history, *n* (%)			
Acute myocardial infarction	76 (25.2)	13 (21.0)	0.48
Diabetes mellitus	132 (43.9)	30 (48.4)	0.51
PCI	68 (22.6)	7 (11.3)	0.045
Dyslipidaemia	27 (9.0)	3 (4.8)	0.28
Hypertension	199 (66.1)	44 (71.0)	0.46
Atrial fibrillation	20 (6.6)	3 (4.8)	0.28
Heart failure	36 (12.0)	5 (8.1)	0.38
COPD	8 (2.7)	0 (0.0)	0.19
Biochemical markers			
White blood cell, ×10^9^/L	7.4 (6.0, 9.7)	11.4 (8.7, 14.4)	< 0.001
Neutrophil, ×10^9^/L	5.4 (3.9, 7.5)	9.3 (6.9, 11.9)	< 0.001
Lymphocyte, ×10^9^/L	1.4 (1.0, 1.8)	1.1 (0.7, 1.5)	< 0.001
Monocyte, ×10^9^/L	0.5 (0.3, 0.6)	0.6 (0.4, 0.9)	< 0.001
Hemoglobin, g/L	132.0 (120.0, 146.0)	128.5 (115.0, 144.0)	0.35
Platelet, ×10^9^/L	199.0 (174.0, 236.0)	189.0 (154.0, 264.0)	0.66
SIRI	1.9 (1.0, 2.9)	4.1 (2.5, 11.7)	< 0.001
eGFR, mL/min/1.73 m^2^	86.0 (66.0, 99.0)	72.0 (42.0, 95.0)	0.005
Troponin I, μg/L	0.2 (0.1, 1.5)	1.1 (0.2, 12.6)	< 0.001
FBG, mmol/L	5.8 (4.8, 8.4)	7.0 (5.2, 10.8)	0.009
HbA1c, %	6.2 (5.8, 7.0)	6.1 (5.7, 7.5)	0.80
TG, mmol/L	1.2 (1.0, 1.7)	1.2 (0.9, 1.6)	0.74
HDL‐C, mmol/L	1.0 (0.9, 1.2)	1.0 (0.8, 1.1)	0.48
LDL‐C, mmol/L	2.5 (1.8, 3.0)	2.5 (2.0, 3.4)	0.091
TyG index	8.8 (8.5, 9.2)	9.1 (8.8, 9.6)	< 0.001
SHR	0.8 (0.7, 1.0)	0.9 (0.7, 1.4)	0.017
PAGln, μmol/L	1929.9 (1560.1, 2477.7)	2334.2 (1884.5, 2703.1)	< 0.001
HMGB1, ng/mL	8.2 (6.3, 10.2)	9.3 (7.9, 11.4)	< 0.001
CH3, μg/mL	49.4 (39.1, 62.6)	57.5 (45.8, 68.4)	< 0.001
LPA, μmol/L	8.2 (6.6, 10.5)	9.2 (7.8, 11.8)	< 0.001
PDGF, ng/mL	115.3 (82.9, 148.2)	142.6 (110.4, 171.7)	< 0.001
Vital sign			
Killip class > II, *n* (%)	34 (11.3)	24 (38.7)	< 0.001
Heart rate, bpm	78.0 (70.0, 88.0)	84.0 (70.0, 94.0)	0.071
Systolic blood pressure, mmHg	131.0 (119.0, 143.0)	117.5 (100.0, 137.0)	< 0.001
Diastolic blood pressure, mmHg	76.0 (70.0, 85.0)	75.5 (58.0, 82.0)	0.081
Left ventricular function			
LVEF, %	55.0 (48.0, 58.0)	48.5 (39.0, 55.0)	< 0.001
In‐hospital treatment, *n* (%)			
Aspirin	270 (89.7)	48 (77.4)	0.008
P2Y_12_ inhibitors	193 (64.1)	39 (62.9)	0.86
β‐blockers	189 (62.8)	32 (51.6)	0.10
ACEI/ARB	175 (58.1)	23 (37.1)	0.002
Statin	283 (94.0)	58 (93.5)	0.89
Anticoagulants	191 (63.5)	47 (75.8)	0.065
PCI	162 (53.8)	27 (43.5)	0.14
GDMT	127 (42.2)	15 (24.2)	0.025

Abbreviations: ACEI/ARB, angiotensin‐converting enzyme inhibitor/angiotensin receptor blocker; CH3, citrullinated histone H3; COPD, chronic obstructive pulmonary disease; eGFR, estimated glomerular filtration rate; FBG, fasting blood glucose; GDMT, guideline‐directed medical therapy; HbA1c, hemoglobin A1c; HDL‐C, high‐density lipoprotein cholesterol; HMGB1, high mobility group box‐1 protein; LDL‐C, low‐density lipoprotein cholesterol; LPA, lysophosphatidic acid; LVEF, left ventricular ejection fraction; MACE, major adverse cardiovascular events; PAGln, phenylacetylglutamine; PCI, percutaneous coronary intervention; PDGF, platelet‐derived growth factor; SHR, stress hyperglycemia ratio; SIRI, systemic inflammation response index; STEMI, ST‐segment elevation myocardial infarction; TG, triglyceride; TyG, triglyceride‐glucose.

### 3.2 Association Between Cluster Phenotypes and MACE

In the SHR quartiles (Q1: 0.06–0.69, Q2: 0.70–0.83, Q3: 0.84–1.07, Q4: 1.08–2.63), higher SHR was associated with higher TyG index and inflammatory marker levels (Table 2). After log‐transformation of biochemical markers, Spearman correlation analysis showed a positive correlation between SHR and inflammatory markers (Table S1). Additionally, abnormal glucose metabolism and inflammatory markers were positively correlated with the SIRI (Table S2).

**TABLE 2:** Inflammatory markers level among SHR quartiles.

Variables	Q1, *n* = 91	Q2, *n* = 95	Q3, *n* = 87	Q4, *n* = 90	*p*
TyG index	8.6 (8.1, 8.9)	8.7 (8.4, 9.1)	9.0 (8.6, 9.5)	9.3 (8.9, 9.5)	< 0.001
PAGln, μmol/L	1872.8 (1533.6, 2252.6)	1929.9 (1550.3, 2557.9)	2100.9 (1713.8, 2477.7)	2322.5 (1633.7, 2691.7)	0.016
HMGB1, ng/mL	8.0 (6.6, 10.2)	8.5 (6.3, 10.4)	8.8 (7.2, 10.2)	9.2 (7.0, 11.2)	0.025
CH3, μg/mL	45.9 (37.0, 61.7)	51.0 (40.5, 64.1)	52.3 (42.8, 64.4)	53.0 (41.4, 64.0)	0.031
LPA, μmol/L	8.0 (7.2, 10.5)	8.4 (6.9, 10.6)	8.6 (6.9, 10.7)	8.8 (6.6, 10.6)	0.024
PDGF, ng/mL	116.9 (80.2, 151.8)	123.0 (87.6, 156.6)	125.0 (83.1, 147.3)	127.2 (92.3, 155.2)	0.038

Abbreviations: CH3, citrullinated histone H3; HMGB1, high mobility group box‐1 protein; LPA, lysophosphatidic acid; PAGln, phenylacetylglutamine; PDGF, platelet‐derived growth factor; SHR, stress hyperglycemia ratio; TyG, triglyceride‐glucose.

**FIGURE 2:**
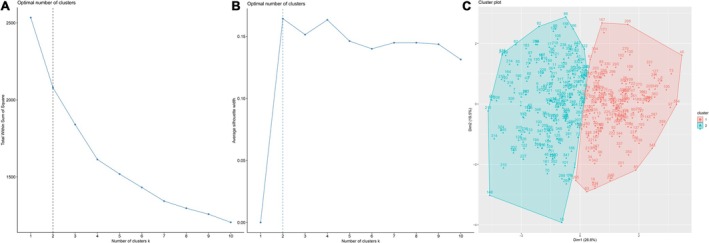
K‐means clustering analysis. (A) Elbow method, (B) Silhouette coefficient, (C) K‐means clustering analysis plot.

K‐means clustering analysis classified patients based on glucose metabolism and inflammation levels. When K = 2, the within‐cluster sum of squares stabilized (Figure 2). Phenotype 2 exhibited higher SHR, TyG index, and inflammatory markers than phenotype 1 (Table S3). Therefore, we labeled the phenotypes as: normal glucose metabolism/low inflammation (class 1) and abnormal glucose metabolism/high inflammation (class 2).

**FIGURE 3:**
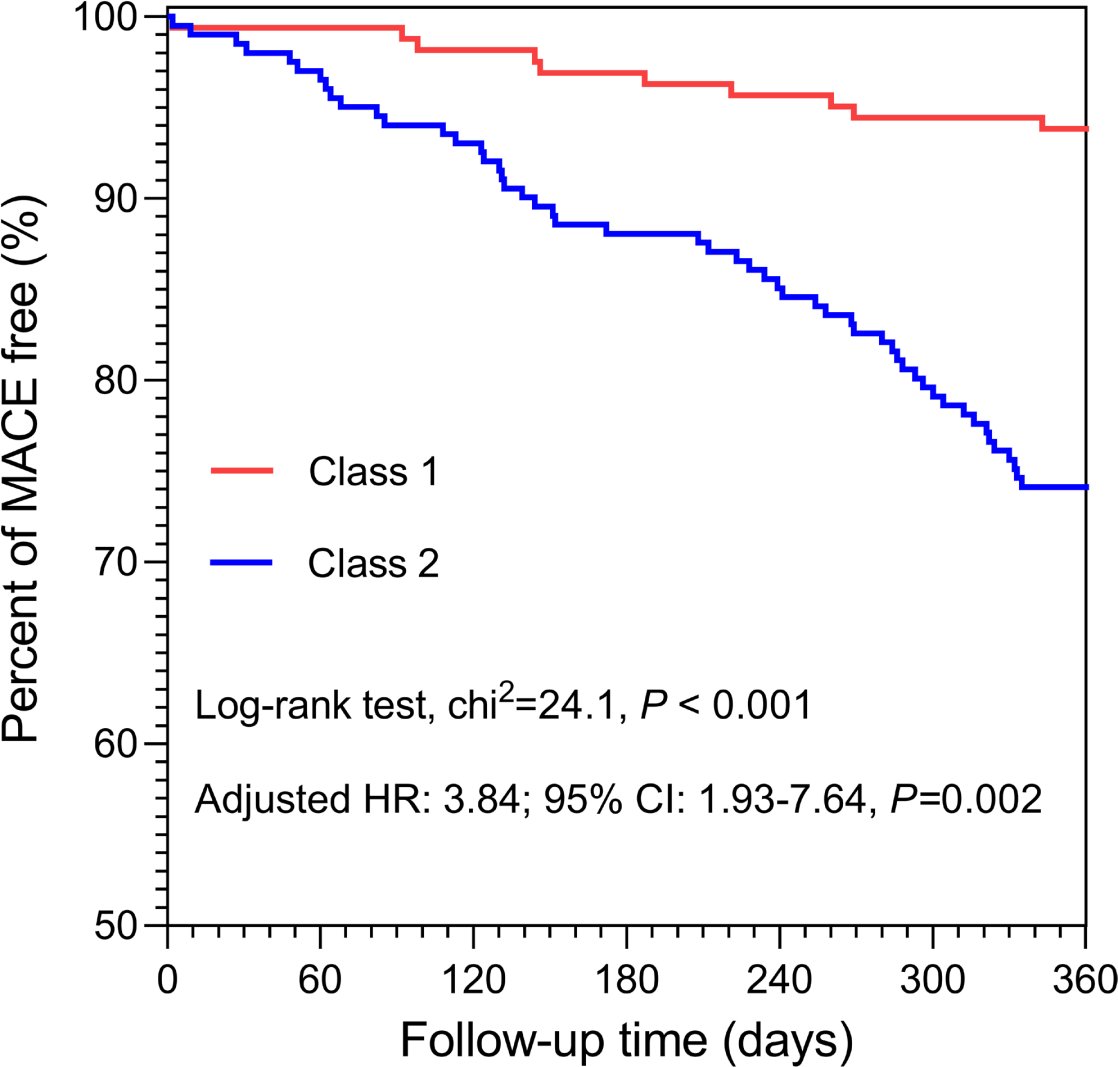
Kaplan–Meier survival analysis. CI, confidence interval; HR, hazard ratio; MACE, major adverse cardiovascular events.

After quartile transformation of SHR, TyG index, HMGB1, PDGF, LPA, PAGln, and CH3, Cox regression analysis showed that these biomarkers were independent risk factors for long‐term MACE, with risk increasing across quartile categories (*p* for trend < 0.001) (Table 3). The abnormal glucose metabolism/high inflammation group had a higher long‐term MACE risk than the normal glucose metabolism/low inflammation group (adjusted hazard [aHR] 3.84, 95% confidence interval [CI] 1.93–7.64). Kaplan–Meier survival curves confirmed a higher MACE incidence during follow‐up in the class 2 group compared to the class 1 group (Figure 3). Moreover, phenotype 2 mediated the effects (14.7%) of left ventricular function on MACE in the mediation analysis (Figure S1).

**TABLE 3:** Association between biomarkers and long‐term MACE.

Quartiles	Crude model		Adjusted model	
	HR & 95% CI	*p*	HR & 95% CI	*p*
SHR				
Q1	Ref.		Ref.	
Q2	2.17 (0.87, 5.37)	0.095	1.74 (0.69, 4.36)	0.239
Q3	2.77 (1.15, 6.67)	0.023	1.82 (0.73, 4.52)	0.196
Q4	4.02 (1.73, 9.32)	0.001	2.53 (1.07, 6.02)	0.035
*p* for trend	< 0.001		< 0.001	
TyG				
Q1	Ref.		Ref.	
Q2	1.23 (0.48, 3.11)	0.664	1.17 (0.46, 2.97)	0.749
Q3	2.57 (1.12, 5.86)	0.025	2.78 (1.20, 6.45)	0.017
Q4	3.43 (1.55, 7.61)	0.002	2.90 (1.28, 6.57)	0.011
*p* for trend	< 0.001		< 0.001	
PAGln				
Q1	Ref.		Ref.	
Q2	6.54 (1.93, 22.2)	0.003	4.66 (1.36, 15.93)	0.017
Q3	7.45 (2.21, 25.07)	0.001	4.76 (1.61, 15.57)	0.014
Q4	7.97 (2.38, 26.72)	< 0.001	5.27 (1.56, 17.88)	0.008
*p* for trend	< 0.001		< 0.001	
HMGB1				
Q1	Ref.		Ref.	
Q2	1.94 (0.77, 4.85)	0.159	2.91 (1.14, 7.43)	0.026
Q3	2.78 (1.16, 6.66)	0.022	3.17 (1.30, 7.71)	0.011
Q4	3.82 (1.65, 8.87)	0.002	3.94 (1.67, 9.28)	0.002
*p* for trend	< 0.001		< 0.001	
CH3				
Q1	Ref.		Ref.	
Q2	1.91 (0.76, 4.80)	0.166	2.21 (0.87, 5.57)	0.094
Q3	2.91 (1.22, 6.93)	0.016	2.39 (0.99, 5.72)	0.051
Q4	3.71 (1.59, 8.64)	0.002	3.16 (1.34, 7.47)	0.009
*p* for trend	< 0.001		< 0.001	
LPA				
Q1	Ref.		Ref.	
Q2	2.75 (1.07, 7.08)	0.036	1.81 (0.69, 4.72)	0.226
Q3	3.32 (1.32, 8.36)	0.011	2.72 (1.09, 6.81)	0.032
Q4	4.43 (1.80, 10.89)	0.001	3.07 (1.20, 7.84)	0.019
*p* for trend	< 0.001		< 0.001	
PDGF				
Q1	Ref.		Ref.	
Q2	1.38 (0.58, 3.26)	0.471	1.34 (0.55, 3.30)	0.518
Q3	2.09 (0.94, 4.65)	0.071	1.92 (0.85, 4.37)	0.118
Q4	2.81 (1.30, 6.07)	0.009	2.54 (1.15, 5.60)	0.021
*p* for trend	< 0.001		< 0.001	
Phenotypes				
Class 1	Ref.		Ref.	
Class 2	4.66 (2.37, 9.16)	< 0.001	3.84 (1.93, 7.64)	0.002

*Note:* Covariates were described in the part of “Statistical Analysis”.

Abbreviations: CH3, citrullinated histone H3; CI, confidence interval; HMGB1, high mobility group box‐1 protein; HR, hazard ratio; LPA, lysophosphatidic acid; MACE, major adverse cardiovascular events; PAGln, phenylacetylglutamine; PDGF, platelet‐derived growth factor; SHR, stress hyperglycemia ratio; TyG, triglyceride‐glucose.

### 3.3 Improvement in GRACE Score Prediction by Cluster Phenotypes

**FIGURE 4:**
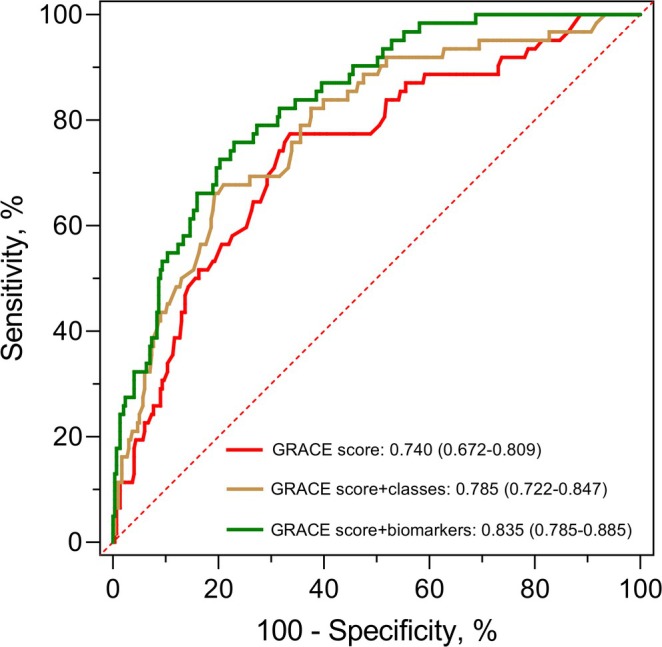
Predictive performance for MACE. GRACE, Global Registry of Acute Coronary Events; MACE, major adverse cardiovascular events.

ROC curve analysis showed that combining the GRACE score with different cluster phenotypes significantly improved its predictive ability for long‐term MACE, with an increased AUC (AUC 0.785, 95% CI 0.722–0.847 vs. AUC 0.740, 95% CI 0.672–0.809; *p* = 0.037) (Figure 4). Furthermore, when biochemical markers of abnormal glucose metabolism and inflammation were incorporated as continuous variables into the GRACE risk score, ROC curve analysis demonstrated a further improvement in its ability to predict MACE (AUC 0.835, 95% CI 0.785–0.885 vs. AUC 0.740, 95% CI 0.672–0.809; *p* = 0.001).

### 3.4 Subgroup Analysis

Subgroup analysis revealed no significant interaction between the abnormal glucose metabolism/high inflammation group and subgroups (age, gender, LVEF, history of acute myocardial infarction, history of PCI, and PCI or not during hospitalization) (*P*_int_ > 0.05) (Figure S2). However, compared to patients who did not receive early GDMT, those who did showed a reduced risk of long‐term MACE (aHR 4.01, 95% CI 2.25–9.64 vs. aHR 2.72, 95% CI 0.61–8.75; *P*_int_ = 0.042).

## 4 Discussion

This study shows a strong association between abnormal glucose metabolism and elevated inflammation. Patients with both abnormal glucose metabolism and high inflammation had a significantly increased risk of long‐term MACE in ACS. These findings highlight the potential biological link between glucose metabolism and inflammation, offering new evidence for early identification and intervention of high‐risk patients.

The results highlighted a strong synergistic effect between abnormal glucose metabolism and heightened inflammation in ACS patients. Abnormal glucose metabolism, characterized by disrupted blood glucose and elevated insulin resistance, weakens insulin's anti‐inflammatory effect, promoting pro‐inflammatory factor release. This triggers a self‐perpetuating inflammatory cycle, increasing systemic inflammation. Endothelial cells in the cardiovascular system, vulnerable to oxidative stress and inflammation, play a crucial role in this process [17, 18]. Dysfunction of these cells disrupts vascular balance, promoting platelet aggregation and coagulation, ultimately increasing the risk of thrombosis and ischemic events.

Among the inflammatory markers, hyperglycemia increases oxidative stress, causing endothelial and myocardial cell damage, which releases HMGB1. HMGB1 binds to RAGE, triggering neutrophil extracellular trap formation and pro‐inflammatory cytokine production, worsening inflammation and promoting MACE [7, 19, 20]. Hyperglycemia also stimulates PDGF secretion, driving smooth muscle cell proliferation and vascular remodeling, which increases plaque instability and thrombosis risk [21, 22]. Additionally, elevated LPA levels, activated by phospholipase A2, reflect inflammation and further promote plaque rupture and thrombosis, contributing to MACE [23]. Moreover, glucose dysregulation leads to gut microbiota imbalance and increased PAGln, which enhances platelet reactivity and smooth muscle function, heightening thrombosis risk and MACE incidence [24].

Therefore, early identification and intervention in patients with abnormal glucose metabolism and high inflammation are crucial for prognosis. While blood glucose control has traditionally been central to diabetes mellitus management, recent insights into inflammation's role in cardiovascular disease have led to incorporating anti‐inflammatory therapies in ACS management. Early intervention improves glucose control, reduces inflammation, and lowers endothelial damage and thrombosis risk. Beyond specific inflammation inhibitors, such as interleukin‐1β inhibition and colchicine, novel hypoglycemic drugs have also been demonstrated to exhibit anti‐inflammatory effects. Notably, a recent animal experiment showed that glucagon‐like peptide‐1 receptor agonist can improve insulin sensitivity in mice, slow down cardiac remodeling by reducing myocardial necrosis, and decreasing the inflammatory response [25]. Moreover, Bray et al. found sodium‐glucose cotransporter‐2 inhibitors significantly reduced inflammatory factors (C‐reactive protein, interleukin‐6) [26]. These findings suggest future trials should evaluate combining novel hypoglycemic drugs and anti‐inflammatory agents in ACS patients with abnormal glucose metabolism/high inflammation (phenotype 2). Additionally, our study shows that early GDMT reduced MACE risk. GDMT, including β‐blockers, ACEI/ARB, statins, and antiplatelet agents, stabilizes plaques, improves myocardial function, and reduces inflammation. In ACS patients with abnormal glucose metabolism and high inflammation, early GDMT can break the cycle of glucose abnormalities and inflammation, protecting endothelial function and reducing thrombosis and ischemic event risk [27].

### 4.1 Strengths and Limitations

This study is the first to use unsupervised machine learning (K‐means clustering) for phenotype classification in ACS, enabling early identification of high‐risk patients with glucose metabolism dysfunction and heightened inflammation. These findings support integrating anti‐inflammatory therapy and hypoglycemic drugs alongside GDMT. However, some limitations exist. First, as a single‐center study, validation through multi‐center, large‐scale cohorts is needed. Second, missing data on high‐sensitivity C‐reactive protein (> 20%) prevented direct correlation analysis with other biomarkers. Given SIRI's validated role in assessing systemic inflammation in ACS by integrating key leukocyte subtypes, we used it as a surrogate. Third, the biological link between glucose metabolism dysfunction and inflammation warrants further investigation. Fourth, K‐means clustering requires pre‐specifying K, and suboptimal selection may introduce bias. It is sensitive to initial centroid selection, often converging to local optima, and its spherical, uniform cluster assumption limits effectiveness for irregular or noisy clinical data. However, we reduced bias by using the elbow method and silhouette coefficient to optimize K selection.

## 5 Conclusions

For ACS patients, especially those with glucose metabolism dysfunction and heightened inflammation, are associated with the higher risk of long‐term MACE. Moreover, early initiation of GDMT is crucial, and the combination of anti‐inflammatory therapies with hypoglycemic agents may further reduce MACE risk.

## Author Contributions

Conception and design of the research: C.Z., W.P., and Y.L. Acquisition of data: C.Z. and W.P. Analysis and interpretation of the data: C.Z., W.P., T.W., M.N., Y.C., and Y.L. Statistical analysis: C.Z., W.P., T.W., M.N., Y.C. Writing of the manuscript: C.Z., W.P., T.W., M.N., Y.C. Critical revision of the manuscript for intellectual content: C.Z., W.P., and Y.L. All authors read and approved the final draft.

## Conflicts of Interest

The authors declare no conflicts of interest.

## Supporting information


**Data S1:** Supporting Information.

## Data Availability

The deidentified participant data will not be shared.
