# Supplementary material for: Association of Abnormal Glucose Metabolism and Inflammation With Prognosis in Patients With Acute Coronary Syndrome
Source: J Diabetes. 2025 Jul 30;17(8):e70134. doi: 10.1111/1753-0407.70134 (PMC12311217; doi:10.1111/1753-0407.70134)
Supplement: Supplementary file 1 — Data S1: Supporting Information. [file JDB-17-e70134-s001.docx]

**Association of Abnormal Glucose Metabolism and Inflammation With Prognosis in Patients With Acute Coronary Syndrome**

**Running Title:** Inflammation, Abnormal Glucose Metabolism, and MACE in ACS

**Content of Supplemental Material**

- **Table S1:** Spearman correlation analysis for SHR and inflammatory markers.
- **Table S2:** Spearman correlation analysis for inflammatory markers and SIRI.
- **Table S3:** Biomarkers level between K-means clustering phenotypes.
- **Figure S1:** Mediating effect of LVEF and MACE associated with phenotype 2.
- **Figure S2:** Subgroup analysis.

**Table S1:** Spearman correlation analysis for SHR and inflammatory markers.

| Variables (Log) | Correlation coefficient | *P* value |
| --- | --- | --- |
| TyG index | 0.42 | <0.001 |
| PAGln, μmol/L | 0.35 | 0.018 |
| HMGB1, ng/mL | 0.27 | 0.021 |
| CH3, μg/mL | 0.14 | 0.028 |
| LPA, μmol/L | 0.12 | 0.031 |
| PDGF, ng/mL | 0.10 | 0.042 |

**Abbreviations:** CH3, citrullinated histone H3; HMGB1, high mobility group box-1 protein; LPA, lysophosphatidic acid; PAGln, phenylacetylglutamine; PDGF, platelet-derived growth factor; SHR, stress hyperglycemia ratio; TyG, triglyceride-glucose.

**Table S2:** Spearman correlation analysis for inflammatory markers and SIRI.

| Variables (Log) | Correlation coefficient | *P* value |
| --- | --- | --- |
| TyG index | 0.13 | 0.024 |
| SHR | 0.25 | <0.001 |
| PAGln, μmol/L | 0.18 | 0.014 |
| HMGB1, ng/mL | 0.19 | 0.011 |
| CH3, μg/mL | 0.15 | 0.021 |
| LPA, μmol/L | 0.23 | <0.001 |
| PDGF, ng/mL | 0.10 | 0.038 |

**Abbreviations:** CH3, citrullinated histone H3; HMGB1, high mobility group box-1 protein; LPA, lysophosphatidic acid; PAGln, phenylacetylglutamine; PDGF, platelet-derived growth factor; SHR, stress hyperglycemia ratio; SIRI, systemic inflammation response index; TyG, triglyceride-glucose.

**Table S3:** Biomarkers level between K-means clustering phenotypes.

| Variables | Class 1 n=162 | Class 2  n=201 | *P* value |  |
| --- | --- | --- | --- | --- |
| TyG index | 8.6 (8.1, 8.9) | 8.7 (8.4, 9.1) | <0.001 | |
| SHR | 0.8 (0.7, 1.0) | 0.9 (0.7, 1.2) | <0.001 |  |
| PAGln, μmol/L | 1850.5 (1548.1, 2410.4) | 2103.3 (1713.8, 2637.5) | 0.002 |  |
| HMGB1, ng/mL | 6.9 (5.5, 8.1) | 10.0 (8.6, 11.4) | <0.001 |  |
| CH3, μg/mL | 41.2 (32.4, 48.5) | 60.9 (49.9, 68.5) | <0.001 |  |
| LPA, μmol/L | 8.3 (7.1, 10.8) | 9.2 (7.4, 11.2) | 0.008 |  |
| PDGF, ng/mL | 87.4 (69.4, 114.5) | 144.3 (121.2, 164.1) | <0.001 |  |

**Abbreviations:** CH3, citrullinated histone H3; HMGB1, high mobility group box-1 protein; LPA, lysophosphatidic acid; PAGln, phenylacetylglutamine; PDGF, platelet-derived growth factor; SHR, stress hyperglycemia ratio; TyG, triglyceride-glucose.


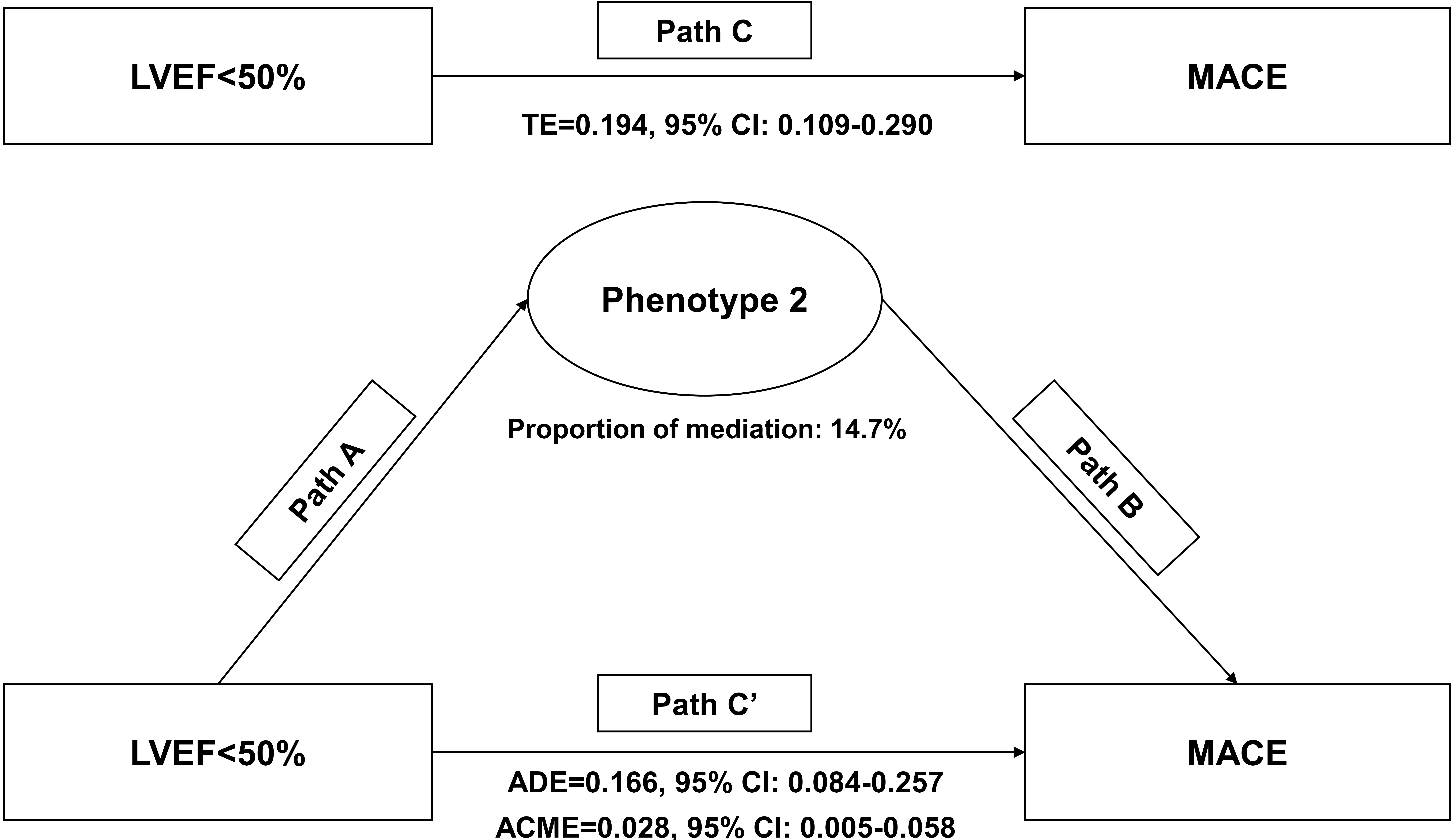


**Figure S1:** Mediating effect of LVEF and MACE associated with phenotype 2. Path A, the effect of LVEF on phenotype 2; Path B, the effect of phenotype 2 on MACE; Path C, the total effect of LVEF on MACE; Path C’, the direct effect of LVEF on MACE after controlling phenotype 2.

**Abbreviations:** ACME average causal mediating effect; ADE average direct effect; CI, confidence interval; LVEF, left ventricular ejection fraction; MACE, major adverse cardiovascular events; TE total effect.


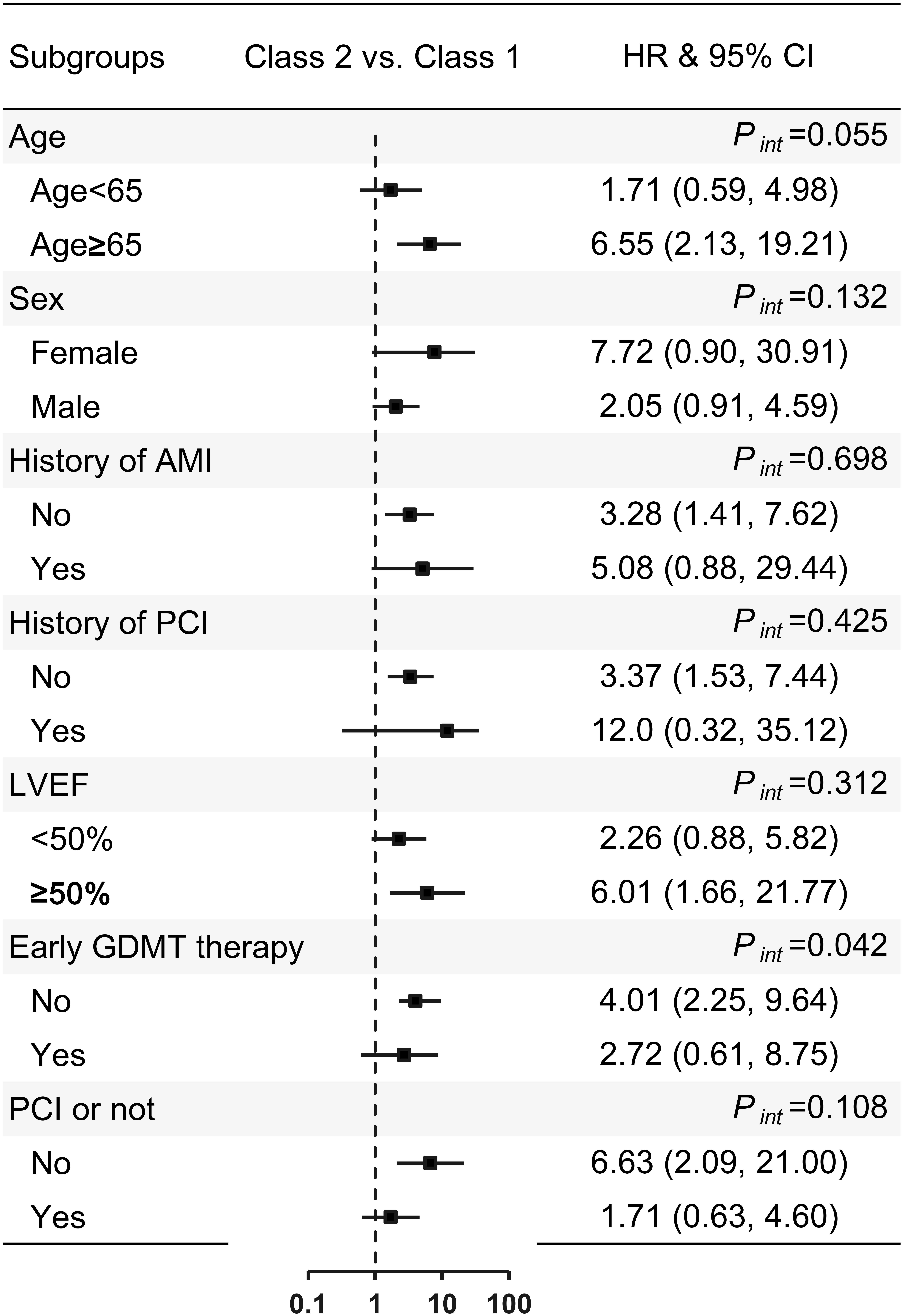


**Figure S2:** Subgroup analysis.

**Abbreviations:** AMI, acute myocardial infarction; CI, confidence interval; GDMT, guideline-directed medical therapy; HR, hazard ratio; LVEF, left ventricular ejection fraction; PCI, percutaneous coronary intervention.
